# Sclerostin in Vascular Calcification: Hypoxia-Driven Regulation and Therapeutic Modulation by Natural Products

**DOI:** 10.1007/s11883-025-01377-w

**Published:** 2026-02-23

**Authors:** Seungyeon Yeon, Sai-Wang Seto, Jyoti Deep Bhuyan, Dennis Chang, Chun Guang Li, Mitchell Low¹

**Affiliations:** 1 NICM Health Research Institute, Western Sydney University, Penrith, NSW 2751 Australia; 2 School of Biomedical Sciences, University of Western Australia, Perth, WA 6009 Australia; 3 Australian Research Council Industrial Transformation Training Centre for Facilitated Advancement of Australia’s Bioactives (FAAB), Sydney, NSW 2109 Australia

**Keywords:** Hypoxia, Hypoxia-inducible factor-1α, Vascular calcification, Sclerostin, Wnt signalling, Natural products

## Abstract

**Purpose of Review:**

Vascular calcification (VC) is increasingly recognized as an actively regulated pathological process rather than passive mineral deposition, strongly associated with aging, atherosclerosis, and chronic kidney disease. Sclerostin, a Wnt signalling antagonist primarily expressed in osteocytes, has recently been implicated in VC, although its precise vascular role remains debated. This review aims to integrate mechanistic and translational evidence on how hypoxia and hypoxia-inducible factor-1α (HIF-1α) regulate sclerostin and contribute to vascular mineralisation.

**Recent Findings:**

Experimental studies demonstrate that HIF-1α activation links hypoxia to key osteogenic pathways, including BMP2, RUNX2, and Wnt/β-catenin signalling, thereby influencing phenotypic switching of vascular smooth muscle cells (VSMCs). Hypoxia exerts both stimulatory and suppressive effects on sclerostin depending on local tissue conditions, reflecting a context-dependent regulatory network. Preclinical and clinical data show that sclerostin can act as either a compensatory inhibitor or a pro-calcific mediator, depending on disease stage and metabolic environment. Emerging evidence further highlights natural products such as polyphenols, flavonoids, and marine-derived compounds that modulate sclerostin expression through oxidative, inflammatory, and Wnt-related pathways.

**Summary:**

Sclerostin sits at a critical intersection between bone and vascular systems, where hypoxia-driven HIF signalling orchestrates its dual effects on mineral metabolism. Understanding the HIF–sclerostin axis provides new insight into the bone–vascular continuum and identifies potential therapeutic targets. Natural bioactive compounds capable of restoring sclerostin–Wnt balance may represent safe, multi-targeted strategies to mitigate VC progression, warranting further mechanistic and translational evaluation.

**Graphical Abstract:**

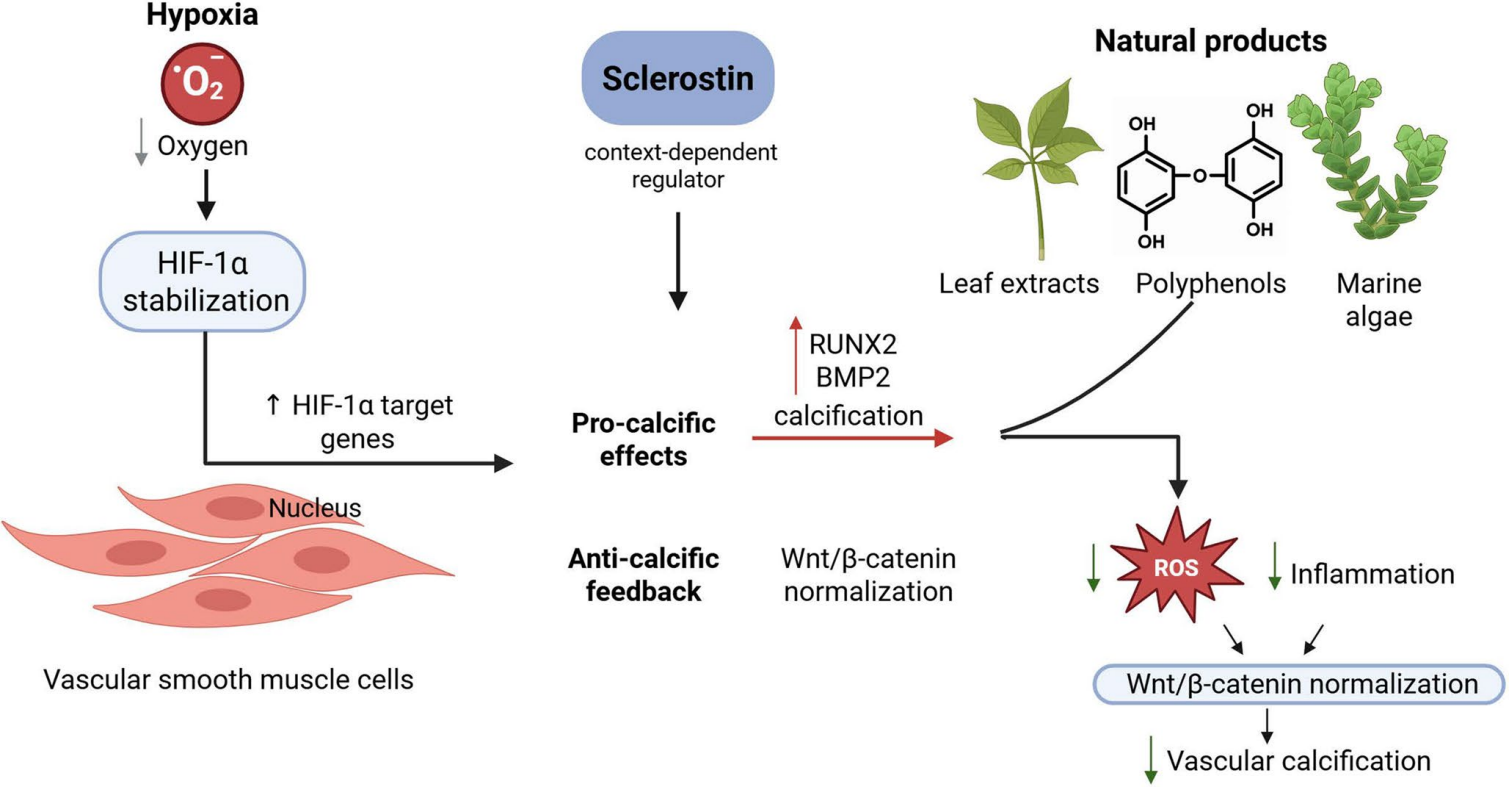

## Introduction

Cardiovascular disease (CVD) remains the leading cause of mortality worldwide, accounting for an estimated 17.9 million deaths annually [1]. Among its underlying mechanisms, vascular calcification (VC) has emerged as a key pathological process that accelerates cardiovascular risk. VC is an actively regulated phenomenon characterised by the phenotypic transition of vascular smooth muscle cells (VSMCs) into osteochondrogenic-like cells [2–4]. It is highly prevalent in ageing individuals and those with chronic conditions such as diabetes mellitus, chronic kidney disease (CKD), and atherosclerosis (AS) [5, 6]. Importantly, VC may develop independently of atherosclerotic plaque formation, indicating distinct pathogenic pathways [7]. Clinically, VC is an independent predictor of cardiovascular morbidity and mortality [8], correlating with increased coronary artery calcium scores, arterial stiffness, and loss of vascular compliance [7, 9, 10].

The concept of VC has shifted from being viewed as a passive degenerative event to an actively regulated, cell-mediated process that resembles bone formation [11]. This transition involves the downregulation of contractile VSMC markers such as transgelin (SM22α), α-smooth muscle actin (α-SMA), and calponin, together with upregulation of osteogenic markers including bone morphogenetic protein-2 (BMP-2), runt-related transcription factor 2 (RUNX2), sclerostin, osteopontin (OPN), osteocalcin, and alkaline phosphatase (ALP) within the vascular wall [12–14].

The bone–vascular axis concept offers a unifying framework for understanding the shared mechanisms of skeletal and vascular mineralisation. In bone, homeostasis is maintained by a balance between osteoblast and osteoclast activity, coordinated through signalling molecules such as BMPs, RUNX2, and sclerostin. Many of these regulators are also expressed in calcified vascular tissue, suggesting common cellular mechanisms underpinning pathological mineral deposition. Current VC research has focused on the dynamic interplay between endogenous inhibitors of calcification such as matrix Gla protein (MGP), pyrophosphate (PPi), and OPN, and pro-calcific mediators including BMP-2 and RUNX2 [15, 16].

Sclerostin is a 213–amino acid secreted glycoprotein encoded by the *SOST* gene and characterised by a modular three-domain architecture [17]. The protein contains an N-terminal signal peptide that directs its secretion, followed by a flexible central loop region, and a highly conserved C-terminal cystine-knot (CTK) domain stabilised by three disulfide bonds [18]. Within the CTK domain, two surface-exposed interaction loops (loop 2 and loop 3) mediate high-affinity binding to the Wnt co-receptors low-density lipoprotein receptor–related proteins 5 and 6 (LRP5/6), thereby inhibiting canonical Wnt/β-catenin signalling [19, 20]. This structural organisation underpins sclerostin’s regulatory role at the bone–vascular interface and may similarly influence VSMC phenotype and pathological vascular mineralisation.

Among these regulators, sclerostin has emerged as a particularly compelling target. Encoded by the *SOST* gene, sclerostin is a glycoprotein initially characterised for its role in inhibiting bone formation through antagonism of the Wnt/β-catenin signalling pathway [20, 21]. Recent studies have demonstrated sclerostin expression in calcified vascular tissues [22, 23], and phosphate-induced sclerostin production in VSMCs suggests that it may act as a compensatory modulator of pathological mineralisation [24, 25]. These findings position sclerostin as a molecular link between skeletal and vascular systems. Circulating bone-related factors, such as osteoprotegerin (OPG) and OPN influence VSMC differentiation and phosphate metabolism [26]. The extent of vascular mineralisation reflects the balance between calcification inhibitors (e.g., MGP, PPi) and pro-calcific drivers (e.g., BMP-2, RUNX2) [27].

The induction of sclerostin by elevated phosphate levels, along with its accumulation in calcified vascular tissues, suggests a potential compensatory mechanism aimed at modulating pathological mineralisation [28–30]. Understanding sclerostin’s dual role in bone and vascular tissues is essential for developing targeted therapies for VC, especially in patients unresponsive to conventional approaches such as phosphate binders or vitamin K supplementation [31, 32]. This review provides an integrated synthesis of current evidence on the mechanistic role of sclerostin in VC. It examines its interactions with key signalling pathways (Wnt/β-catenin, BMP, and HIF) summarises preclinical and clinical findings, and highlights the therapeutic potential of natural products that modulate sclerostin activity. By addressing this underexplored axis of bone–vascular crosstalk, the review aims to clarify key mechanisms and guide future translational research.

## Molecular Mechanisms of Sclerostin in Vascular Calcification

### Wnt Signalling Pathway and Sclerostin’s Role as an Inhibitor

Sclerostin was first identified in the vasculature through proteomic analyses in 2011 and is now recognised to be expressed in the aorta, particularly in VSMCs associated with VC. Both experimental and clinical studies have demonstrated increased sclerostin expression and altered Wnt-signalling components in calcifying vascular tissues [33]. In murine warfarin-induced VC models, sclerostin upregulation attenuates calcification, supporting a protective anti-calcific role [29, 34]. Transgenic *SOST*-overexpressing ApoE^−/−^ mice likewise exhibit reduced atherosclerotic progression and fewer aortic aneurysms through inhibition of canonical Wnt/β-catenin signalling [35]. In vitro, recombinant sclerostin suppresses phosphate-induced calcification in primary human aortic smooth muscle cells (HASMCs), downregulates RUNX2, and restores α-SMA expression. In patients with calcific uraemic arteriolopathy, concurrent upregulation of sclerostin and BMP2 suggests a complex interplay between calcification inhibitors (e.g., MGP) and osteogenic transcription factors [14]. Preclinical work further indicates that elevated circulating sclerostin suppresses BMP2, reinforcing its proposed anti-calcific function [36, 37]. However, the regulatory influence of vitamin D on sclerostin remains inconsistent, with several studies reporting no significant effect on circulating sclerostin levels or vascular outcomes [38].

Clinically, higher circulating sclerostin is frequently associated with VC severity, arterial stiffness, and adverse cardiovascular markers in CKD cohorts [39–43]. This apparent paradox may reflect divergent biological processes in bone versus vascular tissue. Although sclerostin inhibits Wnt/β-catenin–driven osteogenic transdifferentiation in VSMCs, the extreme phosphate–calcium environment in CKD may overwhelm its protective capacity, whereas reduced vascular sclerostin expression in non-CKD cardiovascular disease may permit pathological Wnt activation [39–43]. In type 2 diabetes mellitus (T2DM), elevated circulating sclerostin correlates with increased carotid intima–media thickness and plaque burden [41], and multiple murine models including LDLR^−/−^ mice, Enpp1^−/−^ mice, and diabetic VSMC systems likewise demonstrate increased vascular and systemic sclerostin expression [33, 44, 45]. Despite these associations, the cellular origin of circulating sclerostin in CKD remains unresolved [46].

**Fig. 1:**
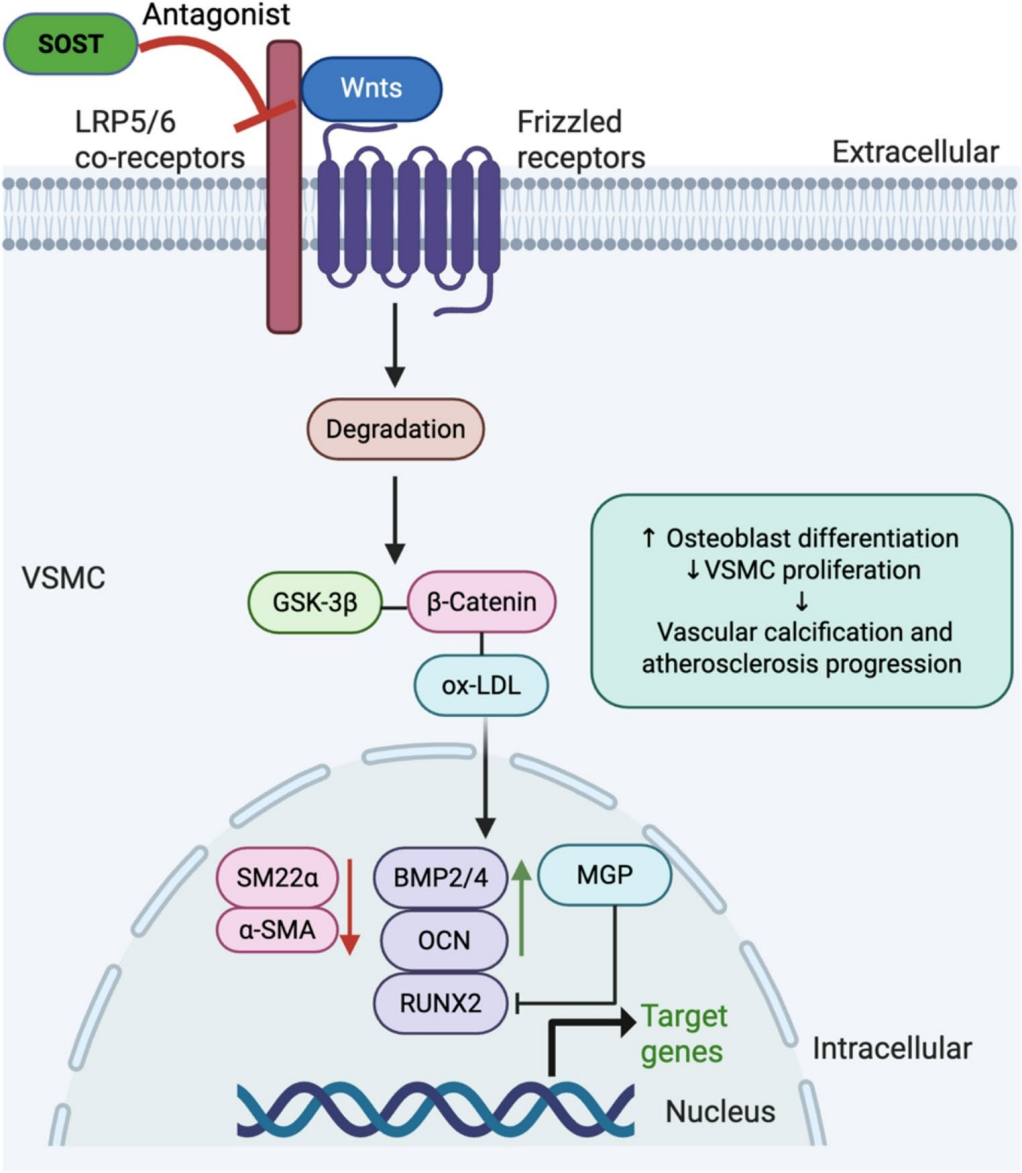
**Mechanisms of vascular calcification: Wnt/β-catenin signalling in vascular smooth muscle cells. **Sclerostin (SOST) binds to low-density lipoprotein receptor–related proteins 5 and 6 (LRP5/6) and antagonizes Wnt ligands, thereby inhibiting canonical Wnt/β-catenin signalling. This suppression prevents β-catenin nuclear translocation and downregulates key osteogenic transcription factors, including bone morphogenetic proteins (BMP2/4), Runt-related transcription factor 2 (RUNX2), and osteocalcin (OCN), ultimately inhibiting osteoblast-like differentiation of vascular smooth muscle cells (VSMCs). These molecular events collectively reduce alkaline phosphatase (ALP) activity and contribute to the attenuation of vascular mineralisation. Arrows indicating biological outcomes (↑/↓) are color-coded to distinguish regulatory effects from signal direction. Figure created with BioRender.com. ***Abbreviations:*** α-SMA, alpha-smooth muscle actin; ALP, alkaline phosphatase; AS, atherosclerosis; BMP, bone morphogenetic protein; GSK-3β, glycogen synthase kinase 3 beta; LRP5/6, low-density lipoprotein receptor–related protein 5/6; MGP, matrix Gla protein; OCN, osteocalcin; RUNX2, runt-related transcription factor 2; SOST, sclerostin; VC, vascular calcification; VSMCs, vascular smooth muscle cells

Mechanistic contradictions across models further complicate interpretation. *SOST*-deficient mice develop more severe VC and cardiovascular abnormalities [47], whereas higher circulating sclerostin in dialysis patients has been associated with less severe aortic valve and aortic wall calcification in several cohorts [48–50]. These discrepancies have important implications for anti-sclerostin therapies such as romosozumab, which, despite its proven efficacy in osteoporosis, has generated cardiovascular safety signals in clinical trials [51, 52]. A growing consensus emphasises that sclerostin’s vascular role is highly context dependent rather than uniformly protective or pathogenic. Elevated circulating sclerostin in dialysis patients may represent a compensatory response, with locally increased expression at calcifying foci acting to restrain pathological Wnt signalling [53]. Conversely, reduced vascular sclerostin expression has been reported in abdominal aortic aneurysm progression, suggesting distinct regulatory dynamics across vascular pathologies [30]. Collectively, these heterogeneous findings underscore the need for standardised, human-relevant experimental systems and longitudinal clinical studies to determine whether sclerostin functions as a pathogenic driver, compensatory modulator, or biomarker of VC. Figure 1 illustrates how sclerostin antagonises LRP5/6-mediated Wnt/β-catenin signalling in VSMCs, thereby limiting osteogenic reprogramming and pathological vascular mineralisation [54].

 Insights into sclerostin’s cardiovascular relevance are further informed by rare hereditary *SOST* loss-of-function disorders, including van Buchem disease and sclerosteosis. Individuals with these conditions exhibit markedly increased bone mass due to lifelong absence of functional sclerostin, yet available case reports and small cohort analyses have not demonstrated a clearly elevated burden of atherosclerotic cardiovascular disease [53]. However, interpretation remains limited due to the extreme rarity of these disorders, small sample sizes, and relatively young age at clinical evaluation. Notably, these observations contrast with the cardiovascular safety signals reported in pharmacologic sclerostin inhibition trials [55, 56], suggesting that congenital deficiency and short-term therapeutic blockade may exert distinct vascular effects. Further integrative genetic, mechanistic, and longitudinal clinical research is needed to clarify the cardiovascular implications of altered sclerostin signalling across different biological contexts. These complexities highlight the importance of understanding how microenvironmental factors, including hypoxia, influence sclerostin expression and vascular responses.

### Hypoxia-Mediated Regulation of Sclerostin

#### HIF Pathway in Vascular Calcification

 The HIF pathway is a central regulator of cellular adaptation to reduced oxygen availability [57]. Under normoxic conditions, HIF-1α is rapidly hydroxylated by prolyl hydroxylase domain (PHD) enzymes and targeted for proteasomal degradation via the von Hippel–Lindau (VHL) E3 ubiquitin ligase complex [58]. However, hypoxic conditions inhibit PHD activity, leading to HIF-1α stabilization, nuclear translocation, and dimerization with HIF-1β (ARNT) [59]. The resulting heterodimer functions as a transcriptional activator, inducing genes involved in angiogenesis, metabolic reprogramming, inflammation, and cell survival [60–62]. These mechanisms are critical not only for maintaining vascular homeostasis but also for promoting pathological processes such as VC.

 The HIF family comprises three α subunits, HIF-1α, HIF-2α, and HIF-3α, with HIF-1α and HIF-2α being the predominant isoforms mediating hypoxia-induced gene expression. Among them, HIF-1α is the most extensively characterized and acts as the master regulator of the hypoxic response [63]. Once stabilized, HIF-1α dimerizes with HIF-1β to form a functional transcription factor that binds hypoxia response elements (HREs) in the promoter regions of target genes. In the setting of VC, HIF-1α activation plays a pivotal role in driving the osteochondrogenic transdifferentiation of VSMCs. This process involves upregulation of osteogenic transcription factors such as RUNX2, BMP2, and SOX9, all of which contribute to calcium deposition and matrix mineralisation. Notably, BMP2 accelerates VC by activating β-catenin signalling and further enhancing RUNX2 expression [64].

 Beyond transcriptional regulation, HIF-1α also orchestrates cellular metabolic reprogramming, a critical component of VC pathophysiology. By inducing glucose transporters (e.g., GLUT1) and glycolytic enzymes, HIF-1α promotes a shift from oxidative phosphorylation to anaerobic glycolysis. This metabolic transition reduces mitochondrial respiration and alters ATP dynamics, fostering a pro-calcific microenvironment that supports osteogenic differentiation and calcific nodule formation in VSMCs.

####  Mechanistic Interplay between Sclerostin and HIF Signalling

 HIFs are central mediators of cellular responses to low oxygen levels, coordinating gene expression programs that influence both osteogenic transformation and VC [65, 66]. Among downstream targets, sclerostin, a potent inhibitor of the Wnt/β-catenin signalling pathway, has emerged as a crucial molecular link between bone metabolism and vascular pathophysiology [67]. The interaction between hypoxia and sclerostin is particularly relevant in VSMCs, where environmental stressors shape the phenotypic transition and calcific potential of these cells [68].

**Fig. 2:**
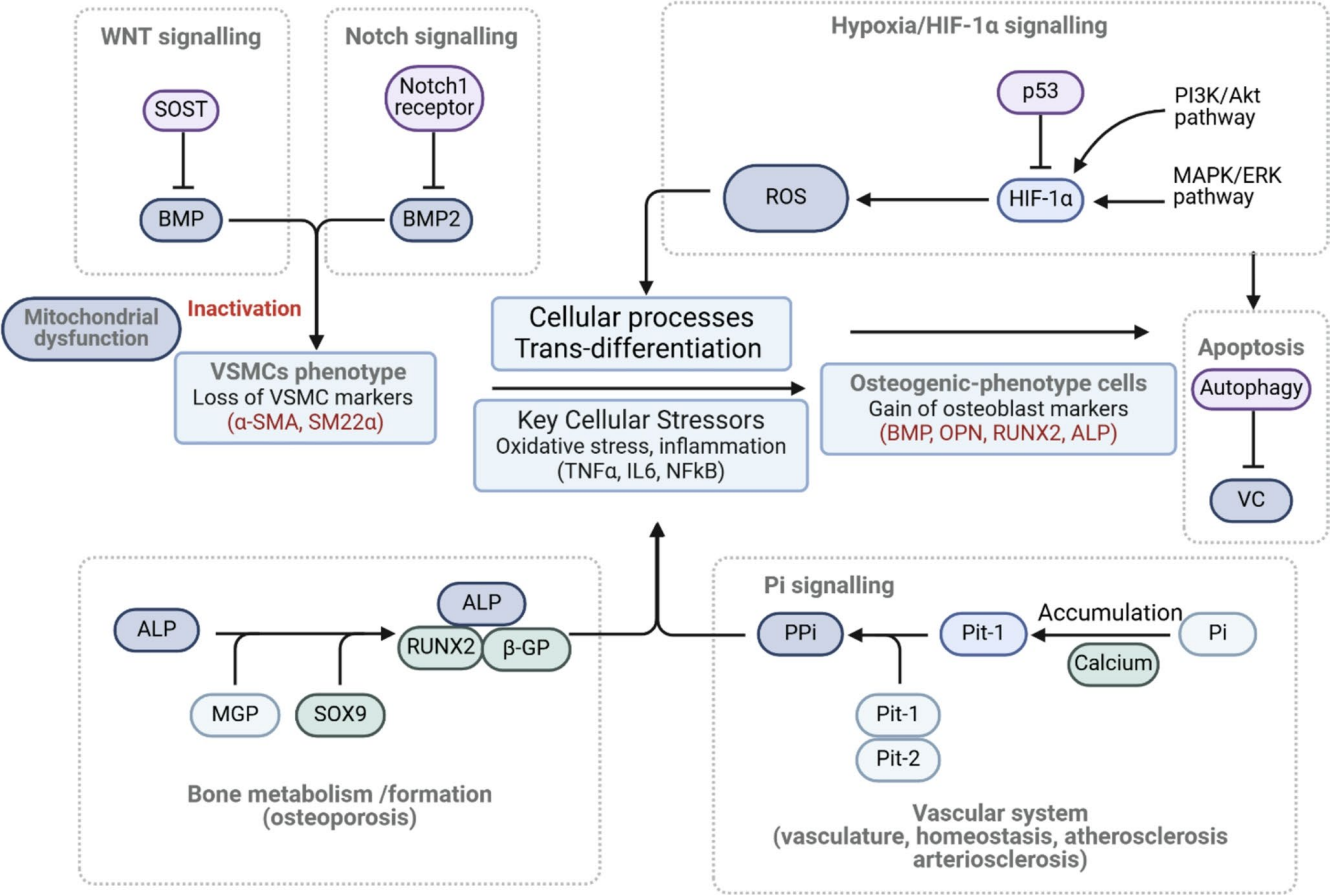
Integrative signalling mechanisms linking bone metabolism and vascular calcification

 Hypoxia regulates sclerostin expression in a tissue- and cell-type–specific manner, reflecting its divergent roles across physiological and pathological conditions. In bone tissue, hypoxia appears to downregulate sclerostin expression through Wnt pathway activation and increased antagonism of BMP signalling, independent of vascular endothelial growth factor (VEGF) activity [65–68]. However, several studies report hypoxia-induced upregulation of sclerostin, potentially via concurrent suppression of Wnt and BMP signalling, resulting in altered osteocyte differentiation [69, 70]. Notably, certain cell types, such as human dental pulp-derived stem cells, exhibit no change in sclerostin expression under hypoxic conditions, highlighting the influence of microenvironmental and lineage-specific factors [66]. To contextualize these dual and model-dependent effects of hypoxia on sclerostin expression and osteogenic signalling, Figure 2 presents an integrative schematic of the molecular pathways involved in VSMC phenotypic modulation. These include HIF-1α, Wnt, BMP, Notch, and phosphate signalling axes, as well as inflammatory and metabolic regulators, all of which converge to promote VC and matrix remodelling.

 This schematic highlights the interconnected molecular pathways involved in regulating vascular smooth muscle cell (VSMC) phenotype, osteogenic transdifferentiation, and calcium deposition. Hypoxia triggers HIF-1α stabilization and reactive oxygen species (ROS) generation, promoting osteogenic markers such as runt-related transcription factor 2 (RUNX2), osteopontin (OPN), and alkaline phosphatase (ALP), while downregulating contractile proteins including alpha-smooth muscle actin (α-SMA) and smooth muscle protein 22-alpha (SM22α). Sclerostin (SOST) acts as a Wnt signalling antagonist, inhibiting BMP activity and contributing to phenotypic modulation. Phosphate (Pi) accumulation via Pit-1/2 and diminished pyrophosphate (PPi) levels facilitate vascular mineralisation. These pro-calcific mechanisms are further influenced by mitochondrial dysfunction and inflammatory mediators including tumor necrosis factor-alpha (TNFα), interleukin-6 (IL-6), and nuclear factor kappa B (NFκB). Elements highlighted in red represent key markers or pathways involved in pathological changes such as osteogenic differentiation and vascular calcification (VC).

 In the vascular system, hypoxia significantly influences VSMC function and calcification processes. Hypoxic conditions enhance VSMC migration, a critical factor contributing to vascular remodelling in pathologies such as aortic stenosis and pulmonary hypertension [71]. Studies have demonstrated that hypoxia promotes calcification in HASMCs, while inhibition of hypoxic signalling attenuates the osteochondrogenic phenotype transition of VSMCs. This regulatory process involves the coordinated action of key proteins, including SOX9, BMP2, and RUNX2, alongside HIF-1α-mediated regulation of glucose metabolism [72–75]. Following the schematic in Figure 2, hypoxia-driven stabilization of HIF-1α also leads to mitochondrial dysfunction and increased production of reactive oxygen species (ROS), which further amplify pro-calcific signalling. These changes are accompanied by suppression of contractile markers such as SM22α and α-SMA, collectively contributing to phenotypic modulation and VC.

 To further delineate these hypoxia-mediated pathways, Table 1 summarizes key molecular regulators and signalling axes involved in VC under hypoxic conditions. This table integrates evidence from various *in vitro* and *in vivo* models, highlighting the diverse mechanisms by which hypoxia influences osteogenic marker expression, calcium deposition, contractile phenotype loss, and inflammation-driven calcification in VSMCs.

**Table 1:** Hypoxia-related mechanisms contributing to vascular calcification

Factors	Effect on VC	Mechanism/Pathway	Experimental Model	References
Estrogen	↓	Reduces BMP2 and RUNX2 levels via HIF-1α signalling	In vitro: rat VSMCs	[74]
Granzyme B	↓	Inhibits store-operated calcium channels under hypoxia; reduces RUNX2, MSX2, BMP2, SOX9; maintains SM22α expression	In vitro: primary rat pulmonary arterial SMCs	[76]
AGEs	↑	HIF-1α/PDK4 pathway increases calcium content, ALP activity, and RUNX2; suppresses glucose metabolism; impaired autophagy worsens calcification	In vitro: rat VSMCs	[77]
Autophagy	↓	Hypoxia-induced AMPK activation promotes autophagy, enhances cell survival, and reduces calcification	In vitro: VSMCs; In vivo: Mice	[78, 79]
Hypoxia	↑	HIF-1α–KLF4 activation promotes abnormal VSMC migration	In vitro: human and rat VSMCs	[71]
		Upregulates RUNX2, SOX9, MSX2, ALP, and OCN; downregulates SMA, calponin, and DMP1 (via HIF-1α pathway)	In vitro: human and rat VSMCs; In vivo: mouse aorta	[73]
		Reduces sclerostin; increases VEGF and IL-8	In vitro: human dental pulp cells	[80, 81]
		Upregulates Notch3 and CaSR; enhances store-operated calcium entry	In vitro: rat VSMCs; In vivo: VHL/PHD2 KO mice; Ang II infusion	[82]
Pi	↑	Induces RUNX2, SOX9, OCN expression; HIF-1α stabilization under hypoxia further enhances osteogenic transdifferentiation	In vitro: rat thoracic aorta VSMCs	[83]
OCN (BGP)	↑	Activates insulin signalling and glucose transporter genes, enhances glucose metabolism	In vitro: mouse VSMCs; In vivo: rat	[84]
Clinical studies				
Hypoxia	↑	Elevated HIF-1α in T2DM patients with coronary artery calcification; NF-κB–HIF-2α activation promotes osteogenic transition and increases VEGF and collagen X	Human coronary arteries; aortic valve tissues	[85]

*Abbreviations*: *AGEs*,* advanced glycation end products; ALP*,* alkaline phosphatase; AMPK*,* AMP-activated protein kinase; Ang II*,* angiotensin II; BGP*,* bone Gla protein; BMP2*,* bone morphogenetic protein 2; BMPs*,* bone morphogenetic proteins; CaSR*,* calcium-sensing receptor; DMP1*,* dentin matrix protein 1; HIF*,* hypoxia-inducible factor; HIF-1α*,* hypoxia-inducible factor-1α; HIF-2α*,* hypoxia-inducible factor-2α; IL-8*,* interleukin-8; KLF4*,* Krüppel-like factor 4; MGP*,* matrix Gla protein; MSX2*,* msh homeobox 2; NF-κB*,* nuclear factor kappa B; Notch3*,* neurogenic locus notch homolog 3; OCN*,* osteocalcin; PDK4*,* pyruvate dehydrogenase kinase 4; Pi*,* inorganic phosphate; PPi*,* pyrophosphate; ROS*,* reactive oxygen species; RUNX2*,* runt-related transcription factor 2; SMA*,* α-smooth muscle actin; SM22α*,* smooth muscle protein 22-α; SOX9*,* SRY-box transcription factor 9; SOST*,* sclerostin; VC*,* vascular calcification; VEGF*,* vascular endothelial growth factor; VHL*,* von Hippel–Lindau; VSMC*,* vascular smooth muscle cell; VSMCs*,* vascular smooth muscle cells. Symbols: ↑*,* upregulation or pro-calcific effect; ↓*,* downregulation or anti-calcific effect*

 While Table 1 outlines key hypoxia-related signalling mechanisms, particularly in diabetes-associated VC, it is important to note that T2DM is a multifactorial disease. Additional contributors such as advanced glycation end-products, chronic inflammation, oxidative stress, impaired autophagy, and disrupted calcium–phosphate metabolism may also influence vascular mineralisation under diabetic conditions [77, 85, 86].

 The interaction between HIF-1α and sclerostin represents a critical axis in vascular pathophysiology, particularly evident in pulmonary hypertension. Chronic hypoxia induces increased sclerostin expression in pulmonary arteries, where BMP-mediated activation of WNT pathways facilitates VSMC proliferation and migration. Concurrently, HIF-1α upregulation modulates sclerostin expression during the osteochondrogenic transition of VSMCs, thereby contributing to VC. These findings highlight the dual regulatory roles of HIF-1α and sclerostin in controlling both cellular phenotype and matrix mineralisation under hypoxic and pro-calcific conditions.

 Mechanistically, the integration of HIF-1α, Wnt/β-catenin, BMP2, and phosphate signalling pathways orchestrates mitochondrial dysfunction, oxidative stress, and transcriptional reprogramming in VSMCs [87, 88]. Hypoxia-inducible HIF-1α dimerizes with HIF-β to form a transcriptionally active complex that drives the expression of pro-osteogenic genes including RUNX2 and ALP [60, 89, 90]. Sclerostin further modulates this axis by antagonizing Wnt signalling, thereby influencing both bone–vascular crosstalk and mineral deposition [34]. As shown in Table 1, multiple regulatory nodes such as Notch signalling, KLF4, autophagy, and calcium-sensing mechanisms also contribute to hypoxia-mediated VC across a range of experimental models.

 In summary, hypoxia promotes VC through the coordinated activation of multiple signalling pathways, including HIF-1α, Wnt/β-catenin, BMP2, and phosphate transport systems. Sclerostin, regulated by both hypoxic stress and osteogenic cues, functions as a variable modulator across disease settings, exerting either protective or pro-calcific effects depending on cell type, disease stage, and local signalling environment. While extensive data exists on the individual roles of sclerostin and HIF-1α, only a few studies have directly assessed both molecules concurrently within the same experimental framework. This gap presents a challenge in fully delineating their potential synergistic or antagonistic interactions and highlights an important direction for future investigation.

 Estrogen is a key endocrine regulator of sclerostin expression. Multiple clinical and experimental studies demonstrate that estrogen therapy reduces circulating and skeletal sclerostin levels, whereas estrogen decline after menopause is associated with increased sclerostin production [91, 92]. Elevated sclerostin in postmenopausal women may contribute to parallel increases in bone turnover and VC susceptibility, suggesting that estrogen deficiency influences the bone–vascular axis through modulation of *SOST* expression [93]. These sex-specific hormonal effects highlight the importance of considering estrogen status when interpreting sclerostin biology, stratifying cardiovascular risk, and evaluating the suitability and timing of sclerostin-targeted or hormone-related therapies.

###  Experimental and Clinical Insights into Sclerostin in Vascular Calcification

 Experimental and clinical studies have provided critical insights into the regulation and functional consequences of sclerostin expression in VC. These findings support the mechanistic concepts introduced in Sections 2.1 and 2.2, particularly regarding the interplay between sclerostin, Wnt signalling, and hypoxia-related pathways. Table 2 summarizes preclinical and clinical data, highlighting how sclerostin expression changes under different conditions and how these changes relate to vascular mineralisation outcomes.

**Table 2:** Mechanisms and regulation of sclerostin in vascular calcification

**Experimental studies on sclerostin in vascular calcification**			
Model (Species – Type)	SOST Expression	Associated Findings / Mechanisms	References
Rat – *in vitro* (β-GP-induced VSMCs)	↑	*Ginkgo biloba* extract attenuates VC; upregulated SOST and downregulated LRP4 inhibit Wnt/β-catenin signalling	[94]
Mouse – *in vitro* (Primary osteoblasts + VSMCs; β-GP + AA)	↑	Upregulated DMP-1, E11, and SOST inhibit Wnt/β-catenin signalling	[33]
Mouse – *in vitro* (Primary VSMCs; 21-day culture)	↑	Long-term culture induces SOST expression during VC progression	
Human – *in vitro* (Pi-treated aortic SMCs)	↓	High phosphate reduced endogenous SOST and increased RUNX2 and BMP2 expression, promoting VC. Treatment with exogenous recombinant SOST restored α-SMA expression and suppressed RUNX2, thereby attenuating calcification	[95]
Rat – *in vivo* (Warfarin-treated aortas and VSMCs)	↑	SOST expression increases in calcified arteries and VSMCs	[34]
Rat – *in vivo* (CKD with high Ca/Pi diet + vitamin D)	↑	SOST is upregulated in CKD-induced vascular calcification	[96]
Mouse – *in vivo* (Warfarin-induced VC; adenine-induced CKD)	↑	SOST protects against VC in both VC and CKD models	[29]
Mouse – *in vivo* (LDLR^−/−^; high-fat diet)	↑	SOST is associated with the BMP2–MSX2–Wnt axis; MSX2 promotes VC	[44]
Mouse – *in vivo* (SOST^Tg^.ApoE^−/−^ transgenic)	↑	SOST overexpression inhibits atherosclerosis progression via Wnt suppression	[35]
Mouse – *in vivo* (STZ-induced T1DM)	↑	Both SOST and β-catenin are upregulated; Wnt signalling is suppressed	[97]
**Clinical studies on sclerostin in vascular calcification**			
Clinical Condition	SOST Expression	Associated Findings/Mechanisms	References
Chronic Kidney Disease (CKD)-Related Conditions			
CKD	↑	SOST inhibits Wnt/β-catenin signalling	[39]
CKD	↑	Serum SOST levels positively correlate with extent of VC	[37, 98–100]
CKD-MBD patients	↑	Increased SOST expression may contribute to development of VC in CKD-MBD	[40]
CKD mineral and bone disorder	↑	SOST is implicated in VC pathogenesis in CKD-MBD	[50, 101]
ESRD	↑	Elevated SOST levels observed in end-stage renal disease	[45]
Renal transplant recipients	↑	SOST inhibits Wnt/β-catenin and attenuates coronary calcification	[102]
Haemodialysis patients	↑	Elevated circulating SOST levels detected	[48]
Haemodialysis + coronary calcification	↑	SOST levels further elevated with coronary artery calcification	[38]
Haemodialysis patients	↑	SOST detected in haemodialysis patients	[103]
CKD (Vitamin D treatment)	→	No change in SOST with vitamin D supplementation	[104]
CKD	↑	Serum SOST levels positively correlated with serum phosphate and inversely with GFR	[105]
Cardiovascular outcomes study	↓	SOST expression reduced or absent in most plaques; no clear association with clinical CVD events	[106]
Non-CKD Vascular Conditions			
T2DM	↑	SOST associated with atherosclerosis and increased intima-media thickness	[41]
T2DM (VSMCs)	↑	Upregulated SOST expression in VSMCs from T2DM patients	[107]
Coronary, aortic, and valve calcification	↑	Elevated SOST levels in multiple calcified vascular beds	[108, 109]
Other Pathways			
Vitamin K– dependent pathway	—	BMP-2–MGP interaction influenced by vitamin K	[110]

*Abbreviations: ApoE, apolipoprotein E; β-GP, β-glycerophosphate; BMP-2, bone morphogenetic protein 2; CKD, chronic kidney disease; DMP-1, dentin matrix acidic phosphoprotein 1; ESRD, end-stage renal disease; *GFR, glomerular filtration rate; *IMT, intima-media thickness; LDLR, low-density lipoprotein receptor; LRP4, low-density lipoprotein receptor–related protein 4; MGP, matrix Gla protein; MSX2, msh homeobox 2; Pi, inorganic phosphate; RUNX2, runt-related transcription factor 2; SOST, sclerostin; STZ, streptozotocin; T1DM, type 1 diabetes mellitus; T2DM, type 2 diabetes mellitus; TGF-β, transforming growth factor beta; VC, vascular calcification; VSMC, vascular smooth muscle cell.Symbols: ↑, increased expression or upregulation; ↓, decreased expression or downregulation; →, no significant change; –, not reported*

 Experimental evidence from *in vitro* and *in vivo* models has consistently demonstrated increased sclerostin expression in calcifying vascular tissues. In rat VSMCs treated with β-glycerophosphate (β-GP), sclerostin upregulation was associated with inhibition of the Wnt/β-catenin pathway, an effect further enhanced by natural compounds such as *Ginkgo biloba* extract [94]. Mouse models also exhibited increased sclerostin expression in both osteoblast–VSMC co-culture systems [33] and long-term VSMC culture models, suggesting that sclerostin may act as a late-stage modulator during VC progression. In contrast, in HASMCs exposed to high phosphate, endogenous sclerostin was downregulated, while RUNX2 and BMP2 were upregulated. Treatment with exogenous recombinant SOST restored α-SMA expression and suppressed RUNX2, thereby attenuating calcification [95], highlighting species- and condition-specific differences. *In vivo* studies using warfarin, high phosphate/calcium diets, and CKD models showed consistent sclerostin upregulation in calcified arteries, often interpreted as a compensatory response with anti-calcific potential [29, 34, 35, 44, 96, 97].

 Clinical studies generally report elevated circulating sclerostin levels in patients with CKD, end-stage renal disease, or those undergoing haemodialysis, with these increases often correlating with the severity or extent of VC [37–40, 45, 48, 50, 98–101, 103]. However, this trend is not consistent across all studies. Several investigations have reported unchanged or even reduced sclerostin levels, depending on factors such as dialysis modality, vitamin D supplementation, or serum phosphate concentration [46, 104]. Others found a positive correlation between serum phosphate and circulating sclerostin levels [105], suggesting a phosphate-driven or adaptive regulatory mechanism. These discrepancies have led to ongoing debate as to whether elevated sclerostin in CKD reflects a causative, compensatory, or merely correlative mechanism. Outside of CKD, increased circulating sclerostin has been associated with arterial stiffness, intima-media thickening, and coronary or valvular calcification in patients with T2DM and aortic stenosis [41, 107–109]. Nevertheless, a dual approach combining immunohistochemical analysis of 144 human atherosclerotic plaques and a large-scale phenome-wide association study involving over 360,000 individuals found no association between reduced vascular sclerostin expression or genetically lowered lifelong sclerostin levels and major cardiovascular outcomes such as myocardial infarction, stroke, or cardiovascular death [106].

 These discrepancies should not be regarded as simple contradictions but rather as condition-dependent regulatory patterns. Species differences are particularly important, as rodent models often show protective upregulation of sclerostin under calcifying stimuli [29, 34, 35, 44, 96, 97], whereas primary human HASMCs exposed to high phosphate frequently display downregulation or functional loss of SOST [95], reflecting differential sensitivity to phosphate and vitamin D pathways. Methodological variability also strongly contributes, since calcification induction protocols (e.g., Pi vs. β-GP), extracellular calcium concentration, culture duration, and the assays used to quantify sclerostin (ELISA, immunohistochemistry, transcriptomics) can produce divergent outcomes [33, 46, 95, 104]. Finally, the disease stage and vascular microenvironment critically shape sclerostin’s role: in early versus advanced CKD, in diabetic versus atherosclerotic vasculature, or across vascular beds with differing hypoxic stress, SOST may act as a compensatory inhibitor, a neutral bystander, or even a pro-calcific mediator [37–41, 45, 48, 50, 98–101, 103, 105, 107–109]. Recognizing these confounders is essential to reconcile apparently paradoxical findings and highlights the need for standardized, human-relevant models that can resolve species- and condition-specific effects in VC, ideally supported by longitudinal human studies with well-characterised metabolic and inflammatory profiles.

## Therapeutic Implications of Sclerostin Modulation

###  Effects of Conventional Cardiovascular Drugs on Vascular Calcification and Sclerostin Regulation

 Several widely prescribed cardiovascular drugs have been investigated for their potential influence on VC and sclerostin regulation. Their effects may arise either through primary pharmacological mechanisms or through secondary off-target pathways.

**Statins.** Primarily prescribed for dyslipidaemia, statins are the most extensively studied for their effects on VC. Experimental studies indicate that they can attenuate osteogenic transdifferentiation of VSMCs and downregulate pro-calcific signalling pathways, including Wnt/β-catenin [111]. However, other *in vitro* and animal models have reported neutral or even pro-calcific effects under certain conditions [112]. Clinically, observational studies remain inconsistent, with variability likely reflecting differences in patient characteristics, statin type and dose, and concomitant therapies [9].

**Beta-blockers and renin–angiotensin system inhibitors**. Beta-blockers, a mainstay therapy for hypertension and heart failure, have limited direct evidence for modulating sclerostin or VC. However, their haemodynamic effects may indirectly influence calcification by reducing vascular wall stress and modifying inflammatory signalling. Similarly, renin–angiotensin system inhibitors, including angiotensin-converting enzyme inhibitors and angiotensin receptor blockers, have demonstrated anti-inflammatory and anti-fibrotic effects in preclinical VC models, although findings from clinical studies remain inconsistent and their influence on sclerostin expression is not well defined [113].

**Bisphosphonates**. Widely used in osteoporosis management, bisphosphonates have been explored for their potential to inhibit ectopic calcification by directly binding to hydroxyapatite crystals and interfering with mineral deposition [114]. While some observational studies suggest that bisphosphonate use is associated with reduced vascular and valvular calcification, others have reported neutral or mixed effects, and their impact on sclerostin expression remains poorly defined [115]. These mixed findings underscore the limitations of conventional drugs in modulating sclerostin and highlight the need for more targeted therapeutic strategies.

###  Anti-Sclerostin Therapeutic Approaches

**Romosozumab** Targeted anti-sclerostin therapies have emerged as a novel approach with potential implications for VC. Romosozumab, a humanized monoclonal antibody against sclerostin, represents a major advance in osteoporosis treatment [116]. By neutralizing sclerostin and enhancing Wnt/β-catenin signalling, it promotes osteoblast activity and bone formation. Randomized controlled trials in postmenopausal women aged 55–90 years showed that romosozumab significantly increased bone mineral density at both the lumbar spine and total hip, with a 73% reduction in new vertebral fractures compared with placebo after 12 months, and superior fracture prevention relative to alendronate in head-to-head trials [117–119].

**Cardiovascular safety** Despite these skeletal benefits, cardiovascular safety concerns have emerged. In the Phase III ARCH trial (Active-Controlled Fracture Study in Postmenopausal Women with Osteoporosis at High Risk), an imbalance in adjudicated serious cardiovascular events, including myocardial infarction, stroke, and cardiovascular death, was reported during the first year of treatment (2.5% in the romosozumab group vs. 1.9% in the alendronate group) [55, 110]. Subsequent meta-analyses and post-marketing surveillance up to 2024 [120], including a recent network meta-analysis of randomized trials and a large real-world cohort study [121], have confirmed that the absolute cardiovascular risk remains low but may be clinically relevant in high-risk populations, particularly those with recent myocardial infarction or stroke. Consequently, major regulatory agencies (FDA, EMA, TGA, and PMDA) restrict romosozumab use to patients at high fracture risk without a recent history of cardiovascular events. Recent pharmacovigilance analyses and longer-term follow-up studies further support a cautious approach to romosozumab use in individuals with established cardiovascular disease, reinforcing the need to balance fracture benefits against potential vascular risk in this population [55, 110, 120–123].

**Small-molecule inhibitors and other bone-active agents** Beyond monoclonal antibodies, small-molecule inhibitors (e.g., F1, VA1, C07) targeting the loop 3 domain of sclerostin–LRP5/6 interactions have been developed [124]. These compounds demonstrate superior tissue penetration and oral bioavailability, and in preclinical VC models have shown osteoprotective effects with potential cardioprotective activity [51, 125]. In addition, conventional bone-active therapies, including bisphosphonates and calcimimetics, have also demonstrated VC attenuation in CKD animal models. Limited clinical data suggest modest benefit in advanced CKD, with bisphosphonates and cinacalcet slowing VC progression by modulating bone turnover and mineral metabolism [126].

**Limitations and translational challenges **While these targeted strategies hold promise, their clinical translation to VC is constrained by several limitations. First, their high treatment costs limit accessibility in many healthcare systems, with reimbursement often restricted to severe osteoporosis cases and treatment duration generally limited to 12 months, requiring sequential antiresorptive therapy thereafter [127–129]. Second, long-term safety beyond approved treatment courses remains uncertain, particularly in patients with CKD, diabetes, or concurrent CVD [130]. Third, dual skeletal-vascular effects raise concerns, as excessive Wnt activation in vascular cells has been shown in preclinical models to exacerbate calcification [131, 132]. Emerging tissue-specific delivery strategies such as vascular-targeted nanoparticles, antibody–drug conjugates, and RNA-based systems could improve efficacy while minimizing off-target skeletal effects [133]. Ultimately, clinical translation will require rigorous cardiovascular safety evaluation, cost-effectiveness analyses, and comparative trials. In parallel, patient-specific factors such as baseline cardiovascular status, renal function, mineral metabolism, and fracture risk should be incorporated into risk–benefit frameworks to guide individualized sclerostin-targeted therapy [134].

###  Natural Products in Sclerostin Regulation

 Natural products have gained increasing attention as complementary or alternative modulators of sclerostin-associated pathways in VC, particularly in the absence of approved pharmacologic therapies that directly target sclerostin. These compounds frequently exhibit multi-targeted biological actions with comparatively favourable safety and cost profiles, making them attractive candidates for long-term vascular protection [135, 136]. Current clinical strategies predominantly address upstream metabolic disturbances, including hyperphosphatemia in CKD, oxidative stress, glycaemic dysregulation, and chronic inflammation, rather than directly modulating the intracellular pathways that regulate *SOST* expression and downstream Wnt or BMP signalling [8, 137–140].

 Polyphenolic compounds such as resveratrol and curcumin have been shown to reduce sclerostin expression and inhibit osteogenic transdifferentiation in β-glycerophosphate–induced VSMCs through modulation of canonical Wnt/β-catenin signaling [141]. In adenine-induced CKD models, resveratrol reduces arterial sclerostin levels, vascular stiffness, and mineral deposition, supporting a mechanistic link between redox regulation and *SOST* modulation. Marine-derived polysaccharides such as fucoid [142–144]. Flavonoids including quercetin, kaempferol, and icariin appear to influence sclerostin indirectly through antioxidant and anti-inflammatory actions, as oxidative stress and cytokine signalling are key drivers of pathological *SOST* upregulation during VC [143, 145–150]. Taken together, these mechanistic actions illustrate the potential of natural products to modulate interconnected upstream triggers of VC rather than acting on a single linear pathway. Table 3. summarises representative natural products, their mechanisms of action, and reported effects on sclerostin regulation.

**Table 3.** Sclerostin regulation by natural products and therapeutic agents in vascular calcification

Compound/Drug	Origin		Dosage & Standardization	SOST Regulation	Study Type/ Experimental Model	Key Findings	Ref.
GBE		*Ginkgo biloba* leaf extract	EGb 761 (24% flavonoid glycosides, 6% terpene lactones); 25–100 µg/mL	↓	*In vitro*: β-GP-induced VSMC calcification	GBE alleviate high phosphorus-induced VSMC VC by inhibiting Wnt/β‐catenin signalling pathway	[94]
Vitamin D		Vitamin/Hormone	1,25(OH)₂D₃ (10–100 nM, 10 ng/kg/day); Calcitriol (0.5 μg/kg, 3×/week)	↓	In vitro: VSMC	_	[151]
				↓	*In vivo*: C57BL/6N mice	SOST inhibited BMP2-induced VC	[36]
					In vitro: VSMC	Downregulated RUNX2 & recover α-SMA activity	
				↑	*In vivo*: CKD rats	High Ca/P diet & vitamin D induced VC	[96]
COX-2/TGF-β		Endogenous protein/cytokine	TGF-β1: 10 ng/mL	↓	In vitro: VSMC	TGF-β1 promoted VC via COX-2 upregulation	[152]
Ginsenoside Rb1*		Panax ginseng root extract	50-100 μM / 20 mg/kg/day, oral gavage	Not specified	*In vitro*: Primary rat VSMCs	Rb1 ameliorated VC by inhibiting Wnt/β‐catenin	[153]
					*In vivo*: CKD rats		
Shuxuetong*		Traditional Chinese medicine	4 mL/kg/day, intraperitoneal injection	↑	In vivo: Rat	Effective against dexamethasone‑induced VC	[154]
APE1/Ref-1*		Endogenous enzyme	Recombinant protein, 1-2 μg/mL	Not specified	*In vitro*: Pi-induced VSMC calcification	Inhibited Pi-induced VC via oxidative stress suppression	[155]
AGE*		Metabolic product	100-200 μg/mL	Not specified	*In vitro*: β-GP-induced VSMC calcification	AGE enhanced HIF-1α expression in VC	[72]

*Abbreviations: AGE, advanced glycation end product; ALP, alkaline phosphatase; APE1, apurinic/apyrimidinic endonuclease 1; BMP, bone morphogenetic protein; Calcitriol, 1,25-dihydroxyvitamin D₃; COX-2, cyclooxygenase-2; GBE, Ginkgo biloba extract; HIF-1α, hypoxia-inducible factor-1α; Pi, inorganic phosphate; Ref-1, redox factor-1; RUNX2, runt-related transcription factor 2; SOST, sclerostin; TGF-β, transforming growth factor-beta; VC, vascular calcification; VSMC, vascular smooth muscle cell; β-GP, β-glycerophosphate*

*Symbols: ↓, downregulation; ↑, upregulation; N/R, not reported*

Standardization information and dosages are critical for reproducibility and clinical translation of natural product research. Studies lacking standardization information or specific dosages are marked with *. Some natural products lack standardization information, which is a critical limitation in current research

 Across these compound classes, several conserved structural features may underpin their biological activity. Flavonoids commonly contain catechol-type B-ring hydroxylation and a conjugated C2=C3 double bond linked to a 4-oxo group, facilitating ROS scavenging and NF-κB suppression [156]. Polyphenolic stilbenes such as resveratrol feature a planar trans-stilbene scaffold and extended π-conjugation, supporting interactions with redox-sensitive transcriptional complexes and attenuation of Wnt-driven *SOST* expression [157]. Marine polysaccharides including fucoidan possess highly sulfated carbohydrate chains that engage extracellular matrix components and BMP receptors, inhibiting BMP2-driven osteogenic signalling. Triterpenoids and related terpenoid scaffolds contain lipophilic multiring structures that facilitate membrane association or receptor modulation [158]. Collectively, these structure activity relationships provide mechanistic plausibility for modulation of the HIF–sclerostin–Wnt axis by chemically conserved motifs of natural products.

 The regulation of sclerostin exhibits both conserved and species-specific features across humans, mice, and rats [159, 160]. Human VSMC models most closely replicate clinical pathophysiology, whereas rodent models, though valuable for mechanistic exploration, often display divergent responses to vitamin D, phosphate burden, oxygen tension, inflammatory signalling, and VEGF-related pathways [132, 161, 162]. These interspecies differences highlight the importance of human-relevant experimental systems, particularly for studying microenvironmental modulators that influence sclerostin expression. Advanced platforms such as vascular organoids, organ on chip systems, and time-resolved multi-omics provide opportunities to replicate human-specific responses, elucidate temporal regulation along the HIF-1α and sclerostin axis, and bridge the translational gap between preclinical findings and clinical application [163–165].

 Based on current evidence, natural products show considerable promise in regulating sclerostin during VC; however, the strength of evidence varies substantially across compound classes. Resveratrol demonstrates moderate confidence, supported by reproducible findings in preclinical CKD models but limited human validation [141]. Fucoidan shows moderate *in vitro* confidence through suppression of BMP2-SOST signalling, though *in vivo* and clinical evidence remains sparse [142–144]. Flavonoids exhibit low to moderate confidence, as their effects on sclerostin appear largely indirect and mediated through redox and inflammatory pathways rather than demonstrated transcriptional regulation. Persistent challenges, including low bioavailability, chemical instability, variability in extract standardization, supraphysiologic dosing in experimental systems, and uncertainty regarding human relevant exposure, continue to limit translational progress.

 To improve clinical relevance, future research should adopt standardized extract preparations, quantify active constituents, optimise dose response assessment, and prioritise human relevant experimental systems such as primary HAVSMCs, vascular organoids, and microphysiological platforms. Rigorous mechanistic studies directly quantifying sclerostin, BMP, HIF related signalling, and Wnt/β-catenin activity will be essential to determine whether natural product modulation of *SOST* can be translated into clinically meaningful strategies for VC.

##  Conclusions and Future Directions

 VC is increasingly recognized as an active, tightly regulated process driven by the osteogenic transdifferentiation of vascular smooth muscle cells. Across experimental and clinical studies, sclerostin emerges as a context-dependent modulator situated at the intersection of Wnt/β-catenin, BMP, Notch, and hypoxia-responsive pathways. Its effects are not uniformly protective or deleterious; rather, sclerostin’s vascular actions depend on phosphate burden, oxygen tension, inflammatory status, and disease stage. These context-dependent outcomes underscore the need for clearer mechanistic resolution and standardized models to determine whether sclerostin functions as a causal driver, compensatory regulator, or biomarker of VC progression.

A unified research framework is required to clarify the temporal, spatial, and pathway-specific regulation of the HIF-1α–sclerostin axis. Future mechanistic studies should incorporate physiologically relevant primary human VSMCs and employ advanced platforms such as vascular organoids, organ-on-chip systems, CRISPR-engineered models, and time-resolved multi-omics to delineate stage-specific responses. Standardization of phosphate–calcium conditions, hypoxic parameters, culture durations, and quantitative assays will be essential to improve reproducibility and to reconcile discrepancies across the literature.

Translational progress will depend on rigorous validation of natural products and other multi-targeted interventions, including detailed compositional profiling, extract standardization, and pharmacokinetic characterization. Strategies to improve bioavailability and tissue-specific delivery, such as nanoparticle systems, prodrug formulations, and vascular-targeted carriers, will be critical for enabling clinical application. Given the cardiovascular safety concerns associated with pharmacological sclerostin inhibition, therapeutic approaches must carefully balance vascular benefits with skeletal integrity, supported by biomarker-guided monitoring and patient-specific risk stratification.

 Ultimately, advancing sclerostin-targeted strategies for VC will require fully integrated mechanistic, translational, and clinical investigations. A standardized experimental framework combined with high-quality clinical studies will be essential for identifying the patient subgroups most likely to benefit from modulation of the HIF-1α–sclerostin axis and for developing safe, effective vascular-protective interventions.

##  Key References

- Ortega MA, De Leon-Oliva D, Gimeno-Longas MJ, et al. Vascular calcification: molecular networking, pathological implications and translational opportunities. Biomolecules. 2024;14(3):275.10.3390/biom14030275.
  - ○ Comprehensive 2024 review integrating Wnt/β-catenin, BMP2, and RUNX2 signaling with translational perspectives; foundational for this review’s conceptual framework.
- Zhang Z, Wang D, Xu R, et al. The physiological functions and therapeutic potential of HIF-1α in vascular calcification. Biomolecules. 2024;14(12):1592. 10.3390/biom14121592.
  - ○ Highlights recent progress on HIF-1α regulation in vascular calcification and links to osteogenic signaling, directly aligning with this review’s mechanistic focus.
- González-Salvatierra S, García-Fontana C, Lacal J, et al. Cardioprotective function of sclerostin in human vascular smooth muscle cells. Cardiovasc Diabetol. 2023;22(1):301.10.1186/s12933-023-02043-8.
  - ○ Demonstrates that sclerostin reduces calcium deposition and apoptosis in VSMCs, supporting its potential protective role in vascular disease.
- Liang X, Li Y, Wang P, Liu H. Key regulators of vascular calcification in chronic kidney disease: hyperphosphatemia, BMP2, and RUNX2. PeerJ. 2024;12:e18063.10.7717/peerj.18063.
  - ○ Identifies major regulatory pathways underlying CKD-associated calcification relevant to phosphate-driven osteogenic transition.
- Ye G, Liu T, Ding C. Bioinformatics analysis of key genes and potential therapeutic agents for vascular calcification in chronic kidney disease. Nucleosides Nucleotides Nucleic Acids. 2024;43(2):1–19. 10.1080/15257770.2024.2423214.
  - ○ Integrates omics-based identification of VC-related targets and compounds, providing a computational foundation for therapeutic development.
- Cheng S-H, Chu W-H, Chou W-H, et al. Cardiovascular safety of romosozumab compared to commonly used anti-osteoporosis medications: a systematic review and network meta-analysis of randomized controlled trials. Drug Saf. 2025;48(1):7–23.10.1007/s40264-024-01475-9.
  - ○ Provides definitive meta-analytic evidence clarifying the cardiovascular safety profile of romosozumab across large-scale RCTs.
- Tsur A, Cahn A, Levy L, Pollack R. Cardiovascular outcomes of romosozumab treatment—real-world data analysis. JBMR Plus. 2025;9(1):ziae146. 10.1093/jbmrpl/ziae146.
  - ○ Real-world study validating cardiovascular outcomes of romosozumab treatment, complementing controlled clinical evidence.
- Chiu S-H, Wu W-T, Yao T-K, et al. Sclerostin and cardiovascular risk: evaluating the cardiovascular safety of romosozumab in osteoporosis treatment. Biomedicines. 2024;12(12):2880.10.3390/biomedicines12122880.
  - ○ Examines clinical and preclinical evidence of sclerostin modulation in cardiovascular contexts, bridging bone–vascular interactions.
- Pan W, Jie W, Huang H. Vascular calcification: molecular mechanisms and therapeutic interventions. MedComm. 2023;4(1):e200.10.1002/mco2.200.
  - ○ Summarizes emerging molecular mechanisms and pharmacological interventions in VC, offering an updated overview of therapeutic strategies.
- Koval A, Mamadalieva NZ, Mamadalieva R, et al. Success and controversy of natural products as therapeutic modulators of Wnt signaling and its interplay with oxidative stress. Antioxidants. 2025;14(5):591. 10.3390/antiox14050591.
  - ○ Comprehensive 2025 review of natural products modulating Wnt/oxidative pathways, directly supporting this article’s natural compound perspective.

## Data Availability

No datasets were generated or analysed during the current study.
